# Association of Circulating Branched-Chain Amino Acids with Cardiovascular Diseases: A Mendelian Randomization Study

**DOI:** 10.3390/nu15071580

**Published:** 2023-03-24

**Authors:** Huan Xu, Xuanyang Wang, Guannan Geng, Xiaoqing Xu, Lin Liu, Yuntao Zhang, Ziqi Wang, Lulu Wang, Ying Li

**Affiliations:** 1 Department of Nutrition and Food Hygiene, School of Public Health, Harbin Medical University, Harbin 150081, China; 2 The First Affiliated Hospital of Harbin Medical University, Harbin 150001, China

**Keywords:** branched chain amino acids, Mendelian randomization, UK Biobank, cardiovascular diseases

## Abstract

Background: There have been reports linking branched-chain amino acids (BCAAs) to the hazard of various cardiovascular diseases (CVDs); however, the causal role of this relationship is still unclear. We conducted a study using bi-directional two-sample Mendelian randomization (MR) with the aim of investigating the possible causal correlation between BCAAs and 13 types of cardiovascular diseases. Methods: The study analyzed data of the largest genome-wide association studies (GWAS) published for the total BCAAs, encompassing isoleucine, leucine, and valine, which were obtained from the UK Biobank, as well as data for 13 cardiovascular endpoints from the MRC-IEU, the FinnGen consortium, and the EBI database. The approach of the primary dissection used became the inverse-variance-weighted (IVW) approach, with additional analyses using the MR-PRESSO global test as well as MR-Egger regression with a view to determining horizontal pleiotropy. Heterogeneity was evaluated by means of Cochran’s Q test. The study also conducted logistic regression dissection for the sake of investigating the correlation between cardiovascular events and serum BCAAs in the UK biobank cohort study. Results: In this study, it was found that individuals with a genetic predisposition to more elevated levels for circulating total BCAAs had a higher hazard of peripheral arterial disease (OR 1.400, 95% CI 1.063, 1.844; *p* = 0.017) in addition to stroke (OR 1.266, 95% CI 1.012, 1.585; *p* = 0.039); circulating valine casually increased the risk of intracerebral hemorrhage (OR 1.760, 95% CI 1.116, 2.776; *p* = 0.015), along with stroke (OR 1.269, 95% CI 1.079, 1.492; *p* = 0.004); genetically predicted isoleucine showed a positive association with peripheral arterial disease (OR 1.466, 95% CI 1.044, 2.058; *p* = 0.027), along with cardioembolic stroke (OR 1.547, 95% CI 1.126, 2.124; *p* = 0.007); furthermore, leucine causally associated with stroke (OR 1.310, 95% CI 1.031, 1.663, *p* = 0.027). In the UK Biobank cohort study, we detected that total BCAAs (OR: 1.285; 95% CI: 1.009, 1.636), valine (OR: 1.287; 95% CI: 1.009, 1.642), and isoleucine (OR: 1.352; 95% CI: 1.064, 1.718) were independently linked to stroke, but not leucine (OR: 1.146; 95% CI: 0.901, 1.458). No such association was found for BCAAs with peripheral arterial disease and intracerebral hemorrhage in the cohort study. Conclusions: In summary, circulating total BCAAs and valine may be causally associated with stroke. The association of BCAAs with other CVD events needs further study.

## 1. Introduction

Branched-chain amino acids (BCAAs), containing valine, leucine, and isoleucine, become crucial amino acids, which are highly prevalent in animals and humans, which can promote protein synthesis. These amino acids are known to impact crucial metabolic pathways and have been linked to several health conditions. Recent researches have indicated an active correlation between the intensified levels of circulating BCAAs and conditions such as type 2 diabetes (T2DM), insulin resistance, obesity, and dyslipidemia; furthermore, MR researches have demonstrated that BCAAs perhaps have a causal part in advancement of obesity and T2DM [1,2,3].

In addition, numerous epidemiological investigations have indicated there exists a link between the intensified levels of BCAAs and an increased hazard of developing cardiovascular diseases (CVDs). A case-control study conducted on middle-aged and elderly Chinese individuals found that higher BCAA levels were linked to a greater susceptibility to CVD [4]. Similarly, a report encompassing 865 patients with acute heart disease demonstrated that increased plasma BCAAs concentrations were linked to higher rates of hospitalization and all-cause mortality for cardiac failure [5]. In addition, in another study of three cohorts of findings, higher levels of isoleucine and leucine at baseline were significantly linked to a higher CVD hazard, including myocardial infarction, stroke, angiogenesis, and angina, after adjusting for confounding variables. However, the association was not replicated in a separate cohort and was weakened after controlling for conventional lipid markers [6]. Moreover, one cross-sectional research involving six hundred and sixty-six individuals including juveniles and adults who were categorized into obese, overweight, or lean categories found that researchers identified high BCAA levels, specifically leucine and valine, as independent indicators of cardiometabolic risk, regardless of BMI status [7].

However, there are some conflicting results regarding the relationship between circulating BCAAs and CVD risk. A cohort study involving 918 elderly males residing in the community found that those with reduced BCAA levels experienced heightened mortality and major cardiovascular events (MACE) [8]. However, a study involving 2023 patients who underwent cardiac catheterization found that elevated BCAA levels provided protection against death and myocardial events [9]. A recent study detected a reduction in concentrations of BCAAs in the plasma and cerebrospinal fluid of rats with ischemic stroke, as well as in the plasma of acute stroke patients compared to controls. Moreover, lower BCAA levels were also correlated with unfavorable neurological outcomes in patients [10].

Therefore, the published observational studies investigating the relationship between circulating BCAAs and CVDs have yielded conflicting results, and whether the relationship is causal and the causal relationship orientation is still unknown. For the purpose of overcoming the limitations of observational studies, researchers have turned to Mendelian randomization (MR) analysis, in which the genetic variants are considered to be the instrumental variables for the sake of establishing potential causal links in both exposures and consequences with less residual confounding and other biases [11]. This research utilized one two-sample bi-directional Mendelian randomization research for the sake of elucidating this etiological connections in both genetically predicted BCAA concentrations and a broad spectrum of CVDs, encompassing peripheral arterial disease (PAD), stroke, angina, coronary artery disease (CAD), cardioembolic stroke, large artery stroke, ischemic stroke, heart failure, myocardial infarction (MI), transient ischemic attack (TIA), small vessel stroke, subarachnoid hemorrhage (SAH), and intracerebral hemorrhage (ICH). Meanwhile, the UK Biobank cohort study was used to verify these associations. To the best of our knowledge, our study is the first and a relatively comprehensive MR analysis on BCAAs and CVDs.

## 2. Materials and Methods

### 2.1. Bidirectional Mendelian Randomization Analysis

#### Data Sources

Summary statistics for the largest GWAS data on total BCAAs, valine, leucine, and isoleucine originated from UK Biobank, with a total of 115,047 to 115,075 cases included in the analysis. These datasets consist of Nightingale Health analysis of the biomarker profiles originating in a half million blood samples within UK Biobank and easily available to the scientific community in 2020. Data regarding 13 CVD endpoints were acquired from the MRC-IEU, the FinnGen consortium, and the EBI database. Detailed information on data sources for instrumental variables associated with other exposures is described in Supplementary Table S1.

### 2.2. Genetic Instrumental Variants for BCAAs

We undertook several measures to ensure the quality of instrumental single nucleotide polymorphisms (SNPs) for our study. Initially, we identified SNPs that exhibited a robust association with BCAAs (*p* < 5 × 10^−8^). Subsequently, we utilized pairwise-linkage disequilibrium (LD) clumping to guarantee the independence of all instrumental SNPs used for our study (window size = 10,000 kb, *R*^2^ < 0.001). SNPs blessed with a minor allele frequency (MAF) below 0.01 were filtered. We employed the mean *F*-statistic to test for weak instrument bias, excluding SNPs with a low statistical power (*F*-statistic < 10). We also conducted a comprehensive look-up of all SNPs utilized in this research in PhenoScanner to investigate any pleiotropic associations with other phenotypes at the genome-wide significance level that may influence our results. We removed three SNP loci in GCKR (rs1260326) [12,13], PPP2R3A (rs34894639), and NEU2 (rs2943652) for their known pleiotropic effect on multiple human complex traits, which could impact our findings. Supplementary Tables S2–S5 provide detailed information on each SNP.

### 2.3. MR Analysis

One bi-directional MR study was carried out with a view to exploring the possible causal association between circulating BCAA levels and CVDs. The principal analysis method employed was the IVW approach, followed by sensitivity analysis methods such as penalized weighted median, weighted median, maximum likelihood, and simple median. Then, a reverse MR analysis was implemented for the purpose of evaluating the causal association between CVDs and circulating BCAAs, and the IVW approach was considered to be the primary analysis approach, along with sensitivity analyses. Furthermore, in order to examine the horizontal pleiotropy possibility in evaluations of single nucleotide polymorphisms (SNPs) in our dissection, MR-PRESSO analysis and MR-Egger regression were performed, after which we removed outlier SNPs with *p*-values below the threshold and ran the MR analysis again. We also assessed the heterogeneity of SNPs using Cochran’s Q test and applied one random-effect IVW pattern in case essential heterogeneity existed in the causal associations among different genetic variants [14].

The Two-sample MR package in R version 4.1.1 was used to conduct statistical analyses on the reported odds ratios (ORs) and the related 95% confidence intervals (CI) for 13 types of CVDs standardized by scaling to one standard deviation (SD) growth in genetically forecast levels of circulating BCAAs [15]. With a view to considering the multiple check (13 CVDs), the Bonferroni method was utilized for adjusting. As for the statistical significance, we set the related threshold at *p* < 0.004, whereas associations with a *p*-value ranging from 0.05 to 0.004 were deemed suggestive.

### 2.4. The UK Biobank Research

#### 2.4.1. Research Population

There exists a large-scale research initiative concerning the UK Biobank, which followed over 500,000 participants aged between 40 and 69. These individuals were enrolled from 22 different assessment centers all over England, Wales, and Scotland from 2006 to 2010. The research aimed to collect prospective data and analyze them for various research purposes. The individuals completed a touch screen questionnaire covering various aspects of their socio-demographic profile, lifestyle, and environment, and provided biological specimens [16,17]. Our study specifically focused on individuals with diabetes, excluding those without serum BCAAs index, cardiovascular diseases prior to diabetes, and non-European races. Ultimately, 8532 participants were included in our study.

#### 2.4.2. Study Endpoints

The endpoints for this study were the first diagnosis of cardiovascular diseases after the onset of diabetes. All the CVD events were defined by their corresponding codes of ICD-10. There exist codes, such as I64, I63, I61, and I60, used to define stroke, while ischemic stroke was distinguished solely by ICD-10 code I63. Myocardial infarction was identified by codes I21–I23 in ICD-10, while coronary artery disease was discerned by codes I20–I25. The peripheral arterial disease was defined as code I73.9. Heart failure was defined as I50. Angina was defined as code I20. Subarachnoid hemorrhage and cerebral hemorrhage were defined by codes I60 and I61, respectively [18,19]. Due to the lack of ICD-10 codes for certain stroke subtypes, only the above nine types of cardiovascular diseases were studied in this UK biobank analysis.

### 2.5. Statistical Analysis

Baseline characteristics were grouped into tertiles of serum BCAA levels for participants. The means and standard deviations (SD) were used to express continuous variables, whereas percentages were employed for the sake of denoting categorical variables. The models of logistic regression were employed for the sake of evaluating 95% CIs and ORs linking cardiovascular events and serum BCAAs. Model 1 regulated for age, sex, drinking status (present, previous, missing, or never), and smoking status (present, previous, missing, or never). Model 2 regulated for additional variables such as deprivation index of Townsend, mean whole annual household earning (>€100,000, €52,000~€100,000, €31,000~€51,999, €18,000~€30,999, <€18,000, and missing), physical activity (high: ≥7.5 MET hours/week, medium: >0, <7.5 MET hours/week, low: no physical movement) [20], qualifications, and BMI. Model 3 deeply regulated for the history of high cholesterol as well as the history of hypertension.

Statistical analysis was conducted in SPSS Statistics 26.0, with all *p*-values considered two-sided and with a statistical significance of *p* < 0.05.

## 3. Results

### 3.1. Association of Circulating Total BCAAs and CVDs in the Bi-Directional MR Analysis

**Figure 1:**
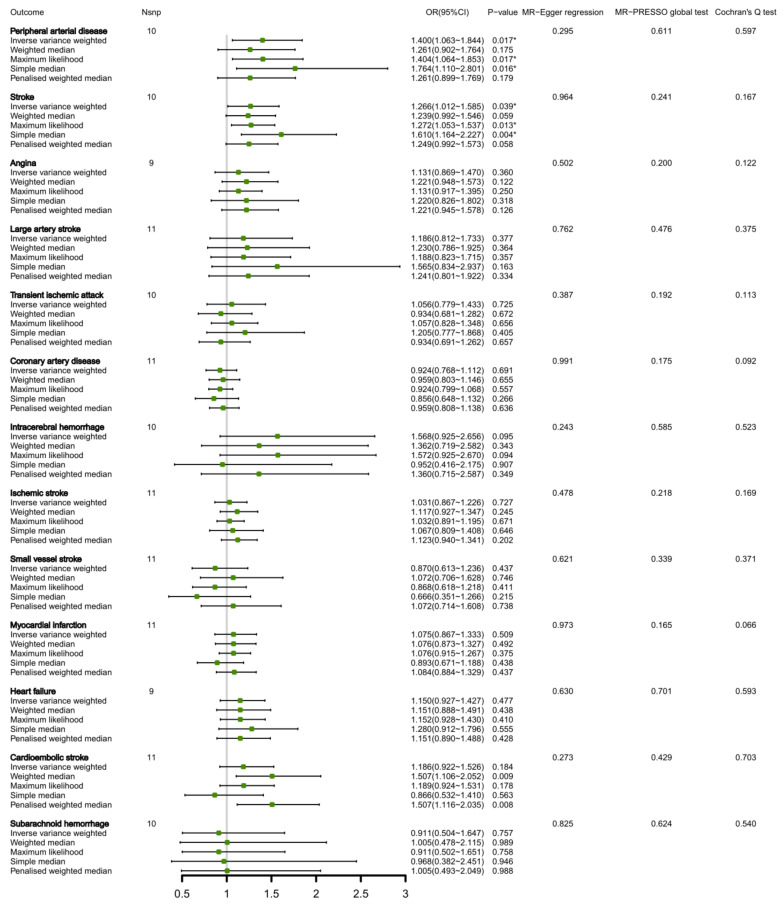
MR estimates the associations between circulating total BCAA levels and CVDs. * Indicates *p* < 0.05. Nsnp, number of SNPs.

A total of 11 SNPs were identified and explained approximately 1.00% variance of circulating total BCAA levels. The details of total BCAAs-related SNPs are shown in Supplementary Table S2. We observed that a genetic predisposition to high circulating total BCAA levels was found to potentially increase the risk of peripheral arterial disease (OR 1.400, 95% CI 1.063, 1.844; *p* = 0.017), and stroke (OR 1.266, 95% CI 1.012, 1.585; *p* = 0.039) according to primary IVW method (Figure 1). Apart from the MR-PRESSO global test, the MR-Egger regression did not yield any indication of directional pleiotropy for the association between total BCAA levels and peripheral arterial disease (*p* = 0.611 for MR-PRESSO; *p* = 0.295 for MR-Egger), along with stroke (*p* = 0.241 for MR-PRESSO; *p* = 0.964 for MR-Egger). Cochran’s Q test indicated no significant heterogeneity exists for these associations. Although the sensitivity analysis weighted median and penalized weighted median showed no statistical association between total BCAAs and peripheral arterial disease and stroke (Figure 1), these methods were employed solely to confirm the effect direction obtained from the primary IVW analysis, rather than establish statistical significance through the *p*-value threshold due to their relatively low statistical power compared to the primary analysis [21]. No significant associations between total BCAAs and other CVDs were observed (Figure 1). No causal effect of the risk of CVDs on circulating total BCAAs was found due to reverse MR analysis (Supplementary Figure S1). Heart failure, transient ischemic attack, small vessel stroke, intracerebral hemorrhage and subarachnoid hemorrhage as exposure factors were excluded from the reverse MR analysis due to their insufficient SNPs (*N* < 3) and limited accuracy that could not explain the corresponding genetic variation effectively.

### 3.2. Connection between Circulating Valine Levels and CVDs by Bi-Directional MR Analysis

**Figure 2:**
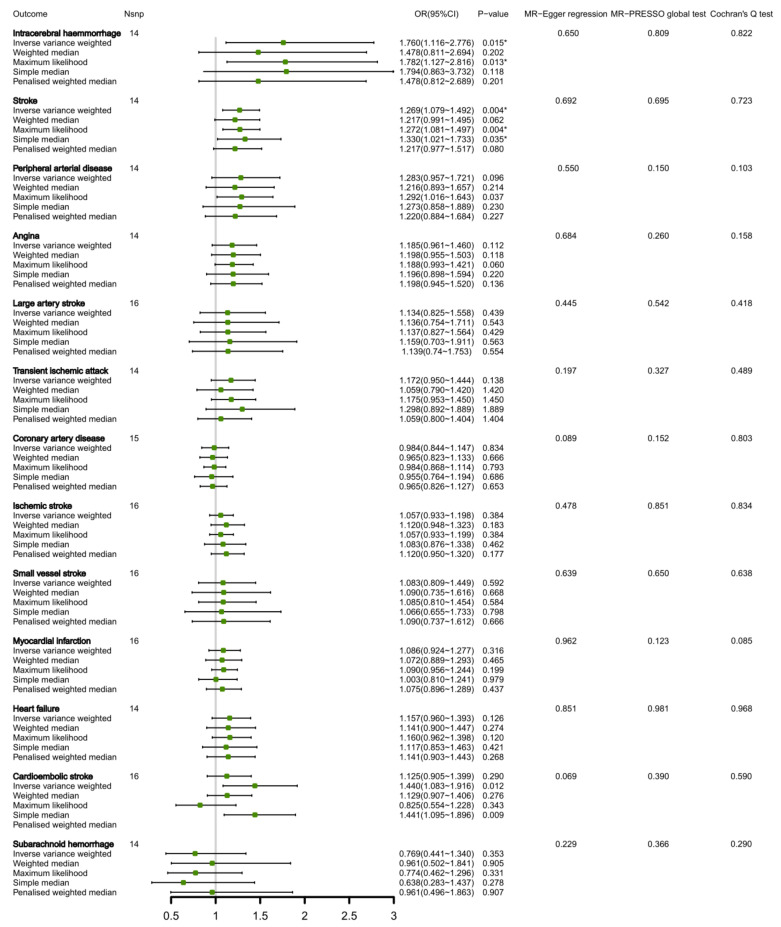
MR estimates of the associations between circulating valine levels and CVDs. * Indicates *p* < 0.05. Nsnp, number of SNPs.

Our genetic analysis showed that 1.4% of circulating valine levels were explained by 16 SNPs, as per Supplementary Table S2. We noticed potential links between the genetic prediction of valine levels and an increased susceptibility to intracerebral hemorrhage (OR 1.760, 95% CI 1.116, 2.776; *p* = 0.015), together with stroke (OR 1.269, 95% CI 1.079, 1.492; *p* = 0.004) due to the IVW method (Figure 2). Aside from MR-Egger regression, MR-PRESSO test did not yield any proof of directional pleiotropy regarding the association of valine levels and either intracerebral hemorrhage (*p* = 0.650 for MR-Egger; *p* = 0.809 for MR-PRESSO), or stroke (*p* = 0.692 for MR-Egger; *p* = 0.695 for MR-PRESSO). Notably, we found no genetically-predicted association between circulating valine levels and other CVDs that were studied in both the primary and supplementary analyses, as displayed in Figure 2. No causal effect of the risk of CVDs on circulating valine levels was found due to reverse MR analysis (Supplementary Figure S2).

### 3.3. Relationship of Circulating Leucine Levels and CVDs Inside Bi-Directional MR Analysis

**Figure 3:**
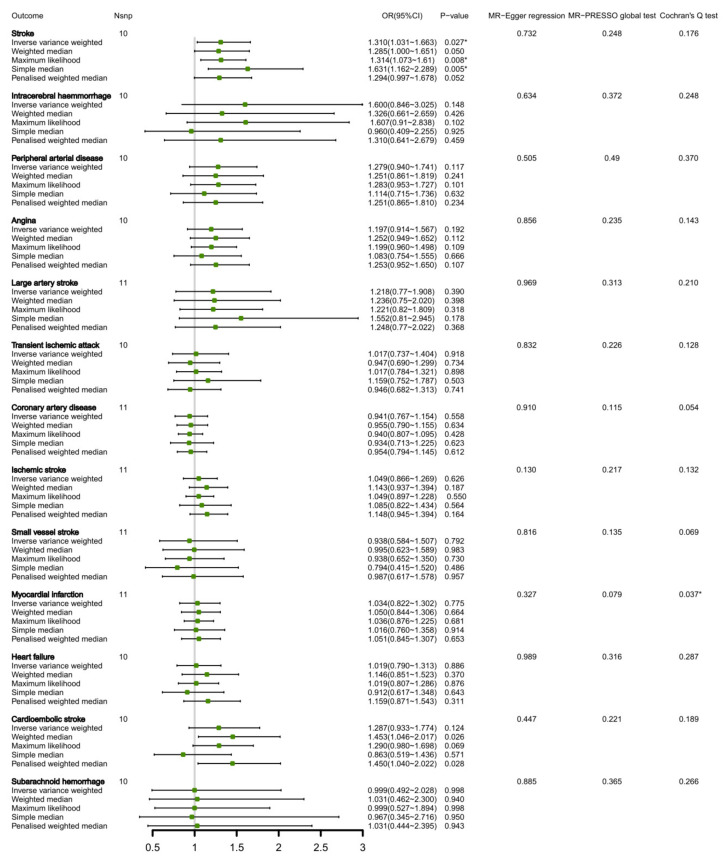
MR estimates of the relationship between CVDs and circulating leucine levels. * Indicates *p* < 0.05. Nsnp, number of SNPs.

We identified 11 SNPs that explained approximately 0.90% variance of circulating leucine levels. Supplementary Table S4 presents detailed information on the leucine-related SNPs. Genetically proxied circulating leucine levels were positively correlated with stroke, with OR equal to 1.310 (95% CI 1.031, 1.663, *p* = 0.027) for each SD growth by using the primary method. Apart from MR-Egger regression, the MR-PRESSO test did not reveal any evidence of pleiotropic effects (*p* = 0.732 and 0.248, respectively). It is worth noting that the causal association between leucine and myocardial infarction exhibited significant heterogeneity according to Cochran’s Q test; however, the random-effect IVW yielded a null result (OR, 1.034; 95% CI, 0.822, 1.302). There was no notable correlation detected between circulating levels of leucine and any other cardiovascular diseases (Figure 3). Reverse MR analysis did not yield significant causal effects of CVDs on circulating leucine levels (Supplementary Figure S3).

### 3.4. Connection of Circulating Isoleucine Levels and CVDs Inside Bi-Directional MR Analysis

**Figure 4:**
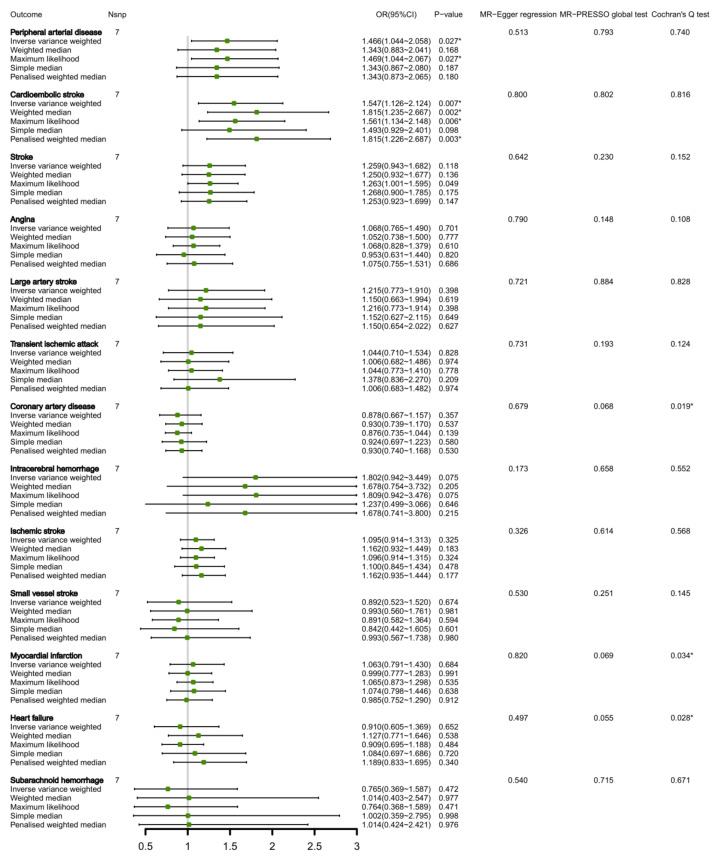
MR estimates of the associations between circulating isoleucine levels and CVDs. * Indicates *p* < 0.05. Nsnp, number of SNPs.

Seven selected SNPs with circulating isoleucine levels explaining approximately 0.61% of the variation are shown in Supplemental Table S5. Figure 4 demonstrates a positive correlation between genetically predicted circulating isoleucine concentrations and peripheral artery disease as well as cardioembolic stroke, with odds ratios of 1.466 (95% CI 1.044, 2.058; *p* = 0.027) and 1.547 (95% CI 1.126, 2.124; *p* = 0.007), respectively. Apart from MR-PRESSO analysis, MR-Egger regression did not provide any horizontal pleiotropy evidence in these associations for either peripheral artery disease (*p* = 0.793 and 0.513, respectively) or cardioembolic stroke (*p* = 0.802 and 0.800, respectively). While Cochran’s Q test revealed significant heterogeneity for the connection between circulating isoleucine and myocardial infarction and heart failure, the random-effect IVW model failed to establish any significant associations (as depicted in Figure 4). Reverse MR analysis did not yield significant causal effects of CVDs on circulating isoleucine levels (Supplementary Figure S4).

### 3.5. The UK Biobank Cohort Study of Circulating BCAAs with the Incidence of CVDs

Supplementary Tables S6–S9 show the baseline characteristics of the 8532 participants by tertiles of baseline measurements of BCAAs metabolites. Table 1 and Supplementary Tables S10–S13 presented the association of circulating BCAAs, valine, leucine, and isoleucine levels with the incident CVD events. As Table 1 shows, Model 3 was used to compare the highest and lowest tertiles, and the results indicate that total BCAAs (OR: 1.285; 95% CI 1.009, 1.636), valine (OR: 1.287; 95% CI 1.009, 1.642), and isoleucine (OR: 1.352; 95% CI 1.064, 1.718) were independently related to the risk of stroke, but not leucine (OR: 1.146; 95% CI: 0.901, 1.458). However, no notable links were found between BCAAs and other CVD events (Supplementary Tables S10–S13).

## 4. Discussion

In this expansive investigation of human genetics, utilizing a genome-wide approach, elevated levels of circulating total BCAAs polymorphisms were found to be linked with an increased susceptibility to peripheral arterial disease and stroke, circulating valine casually increased intracerebral hemorrhage and stroke, genetically predicted isoleucine levels displayed a suggestive positive correlation with both peripheral arterial disease and cardioembolic stroke, and leucine causally associated with stroke. In the UK Biobank cohort study, we detected that total BCAAs, valine, and isoleucine were independently associated with stroke, but not leucine.

Recent research indicates that BCAAs have emerged as promising biomarkers for identifying and targeting CVD-related risk factors. Elevated serum BCAA levels have already been closely linked to intensified CVD incidence. Various investigations have already proven an active connection between circulating BCAA levels and development of insulin resistance and type 2 diabetes that are hazard elements of CVDs [22,23]. Additionally, elevated BCAA levels have already been associated with other CVD hazard factors, including obesity [24], hypertension [25], dyslipidemia [26], and carotid intima-media thickness [27]. These studies suggested that BCAA may be associated with the risk of CVDs.

There is little prospective research investigating the potential link between BCAAs and the risk of CVD. One such study involved a cohort of 27,041 women who had their plasma BCAA metabolites measured in baseline and tracking them for an average of 18.6 years [28]. After adjusting confounders, total BCAAs were positively associated with CVD. BCAAs were found to be linked to myocardial infarction and revascularization, with a borderline significant association with stroke. These associations were greater when T2DM preceded the CVD events. Studies on the relationship between BCAAs and T2DM are consistent. The reason may be that increased BCAA metabolites reflect impaired catabolism and circulatory accumulation, and raise CVD risk by promoting insulin resistance-mediated atherosclerosis [13]. In a case-cohort research concerning a PREDIMED Mediterranean Diet test population, BCAA measurements were obtained from baseline blood samples of 970 high-risk individuals for CVD. A total of 226 cardiovascular events occurred after follow-up. After controlling for potential confounding variables, researchers discovered that elevated isoleucine and leucine levels at baseline were linked to a greater risk of cardiovascular disease, especially stroke. Specifically, a growth of one SD in baseline concentrations of valine, leucine, and isoleucine was associated with elevations of 37%, 45%, and 51% in the hazard of stroke, respectively [29].

These reports align with our findings. We came to the conclusion that circulating total BCAAs, valine, and leucine are positively linked with stroke, and isoleucine is positively correlated with cardioembolic stroke by MR analysis. The UK biobank cohort study showed that total BCAAs, valine, and isoleucine were independently correlated with stroke, but not leucine.

Data on peripheral artery disease and intracerebral hemorrhage are sparse. In our study, genetically predicted elevated circulating total BCAA levels and isoleucine were linked to an intensified hazard of peripheral artery disease; additionally, valine was found to have a causal association with intracerebral hemorrhage. However, the observation study failed to demonstrate a clear pattern of associations between BCAAs and peripheral artery disease and intracerebral hemorrhage. This was possibly due to there being confounding factors in observational studies, whose causal inference ability is inferior to MR analysis.

Recent research illustrated that BCAAs consumption may elevate the hazard of thrombosis due to their ability to promote platelet activation, in which valine and isoleucine may play a more significant role in regulating platelet activation than leucine. BCAA catabolism is facilitated by various mitochondrial enzymes and results in production of BCKAs and acyl-CoAs, which are intermediate metabolites [1,26]. While all three BCAAs are known to promote platelet activation, the ketoacid metabolites of valine and isoleucine (α-ketoisovaleric acid and α-keto-β-methylvaleric acid, respectively) exhibit an more energetic effect on platelet activation than the ketoacid metabolite of leucine (ketoisocaproic acid) [30]. Additionally, propionyl-CoA, a shared metabolite of α-ketoisovaleric acid and α-keto-β-methylvaleric acid, significantly increased platelet activity, suggesting that the metabolic pathway of valine and isoleucine is the main pathway of platelet activation and thrombosis. Moreover, elevated BCAA levels can also activate mTOR, leading to changes in protein transport, lipid metabolism, glucose metabolism, nucleic acid metabolism, and autophagy regulation in the heart [29].

Inside individuals’ intestine, BCAAs are metabolized by specific bacteria, including the genera *Clostridium* and *Bacteroides*, resulting in the formation of branched short-chain fatty acids (BCFAs) such as isovalinic acid, isobutyric acid, and 2-methylbutyric acid that originate in undigested proteins in the colon [31]. Unlike linear forms of the short chain fatty acid, BCFAs do not participate in carbohydrate metabolism [32]. BCFA levels are increased between the proximal colon and distal colon, along with feces, which is considered as a biomarker of colonic protein fermentation. The Western diet, which is low in complex carbohydrates and high in protein, can elevate BCFA levels [31]. Dietary supplementation with complex carbohydrates capable of reaching the colon was able to reduce fecal BCFA levels, whereas protein supplementation increased BCFA levels [33,34]. It has been shown that age is a variable with a clear positive correlation with the molar proportions of BCFA [35]. In addition, BCFA has been implicated in regulating glucose and lipid metabolism. BCFA levels are higher in feces of hypercholesterolemic patients, and serum lipid indicators are worse in patients with high fecal isobutyric acid levels [36]. In both in vitro and in vivo studies, BCFA was found to inhibit the processes of insulin-stimulated lipogenesis and camp-mediated lipolysis, whereas researchers observed isobutyric acid to increase insulin-stimulated glucose uptake [37]. In addition, branched C5:0 was positively correlated with lipid metabolism indicators [38].

Some studies have found that BCAAs may be potential biomarkers in CVD patients through metabolomics analysis. Shah et al. analyzed the plasma of 2023 patients with cardiac catheterization and found that BCAAs were related to mortality [9]. Bhattacharya et al. surveyed an active relationship between coronary artery disease and BCAAs in a study of 1983 cardiac catheterization patients [39]. However, neither of these studies adjusted for traditional lipid measures. In a study with three discovery cohorts, the authors used nuclear magnetic resonance metabolomics to identify biomarkers of CVD and discovered that isoleucine and leucine baseline levels were significantly linked to CVD risk. However, this result was not reproducible in an independent cohort and was weakened after adjustment for traditional lipid markers [6]. According to the results of our study, total BCAAs and valine have causal relationships with stroke, so they may able to be used as biomarkers for the diagnosis of stroke. However, it is worth noting that CVD is just one complicated ailment that is caused by various factors. Accurate diagnosis of CVD requires a comprehensive assessment of the patient’s overall health status, including lifestyle factors and family history [40]. In addition, BCAA levels are not specific for CVD and can be elevated in other diseases such as diabetes and obesity. Therefore, although combining BCAA levels with other parameters has the potential to improve CVD diagnosis, further studies are required to determine the optimal parameter combination and its predictive value in identifying CVD risk. It is also crucial to think about the possible risks and benefits of using these parameters during clinical practice and to develop appropriate guidelines for their use.

MR is just a sophisticated method for causal inference in which the genetic variations (e.g., SNP) are regarded as instrumental variables (IVs) for the purpose of surveying the causal correlation between both exposure factors and consequence phenotype. This methodology leverages genome-wide association studies (GWAS) to gather figures concerning exposure factors and outcome variables, thereby surmounting the limitations of observational studies [41,42]. To ensure the validity of MR, it is crucial to satisfy three fundamental assumptions: First, genetic variants should be highly associated with exposure factors; second, the types of variants need to be unrelated to confounding elements linked to exposure and outcomes; eventually, the genetic variants only have to impact the consequence by means of exposure factors, without any influence from other biological pathways.

Our study utilized an MR analysis approach to detect the possible causal relationship between BCAA levels and hazard of various CVDs. The study leveraged the UK Biobank’s most extensive and up-to-date GWAS database for total BCAAs (*N* = 115,047 cases), valine (*N* = 115,048 cases), leucine (*N* = 115,074 cases), and isoleucine (*N* = 115,075 cases) to yield high-powered estimates of causal association. SNPs characterized by strong genome-wide associations (*p* < 5 × 10^−8^) as well as independent inheritance without any LD were chosen for IVs in this study. Furthermore, to avoid weak instrument bias, we ensured that *F* statistics were well above the threshold of 10. Moreover, we prudently excluded three SNPs, namely rs1260326 in GCKR, rs34894639 in PPP2R3A, and rs2943652 in NEU2, owing to their known pleiotropic effects on a range of human traits, including T2DM, coronary artery disease, BMI, and triglycerides, as evidenced by PhenoScanner database searches [12,13,43]. To enhance the reliability of our findings, sensitivity analyses were implemented by means of weighted median, maximum likelihood, and simple median. These alternative methods used in this study were not as statistically powerful as the primary IVW analysis. However, they were only employed to verify the effect estimates obtained through the primary analysis, rather than solely relying on *p*-value thresholds to determine statistical significance [21].

This investigation has several limitations that should be acknowledged. First, our dissection was limited to examining the connection between CVDs and BCAAs in individuals with diabetes within the UK Biobank cohort study, and the sample size was relatively marginal and could not represent all people. Second, due to the lack of ICD-10 codes for certain stroke subtypes, our study was restricted to only nine types of cardiovascular disease in an observation study. Finally, our dissections largely originated in European people who are not perhaps entirely applicable to other ethnic populations.

To sum up, this research found proofs that the genetic predisposition to elevated circulating BCAA levels may be at increased risk for peripheral arterial disease and stroke, circulating valine casually increased intracerebral hemorrhage and stroke, genetically predicted higher levels of isoleucine could increase risk of developing peripheral arterial disease and cardioembolic stroke, and leucine was causally associated with stroke. In a separate study involving the UK Biobank cohort, we detected independent associations between total BCAAs, valine, and isoleucine with stroke, but not peripheral arterial disease and intracerebral hemorrhage. Taken together, circulating total BCAAs and valine may be causally associated with stroke. The association of BCAAs with other CVD events merits further study.

## Figures and Tables

**Table 1:** Association of baseline circulating BCAAs and the risk of multiple cardiovascular diseases.

Serum Total BCAA Levels (mmol/L)				
	Tertile 1 (<0.346)	Tertile 2 (0.346~0.416)	Tertile 3 (>0.416)	
	Prevalence ratio	Prevalence ratio	Prevalence ratio	*p*-trend
Stroke	(95% CI)	(95% CI)	(95% CI)	
Model 1	Reference	1.188 (0.935~1.510)	1.290 (1.017~1.636)	0.038 *
Model 2	Reference	1.190 (0.935~1.516)	1.293 (1.106~1.646)	0.039 *
Model 3	Reference	1.188 (0.933~1.514)	1.285 (1.009~1.636)	0.044 *
Serum valine levels (mmol/L)				
	Tertile 1 (<0.203)	Tertile 2 (0.203~0.238)	Tertile 3 (>0.238)	
	Prevalence ratio	Prevalence ratio	Prevalence ratio	*p*-trend
Stroke	(95% CI)	(95% CI)	(95% CI)	
Model 1	Reference	1.244 (0.981~1.577)	1.278 (1.007~1.623)	0.047 *
Model 2	Reference	1.244 (0.978~1.581)	1.284 (1.006~1.638)	0.049 *
Model 3	Reference	1.245 (0.979~1.584)	1.287 (1.009~1.642)	0.047 *
Serum leucine levels (mmol/L)				
	Tertile 1 (<0.096)	Tertile 2 (0.096~0.119)	Tertile 3 (>0.119)	
	Prevalence ratio	Prevalence ratio	Prevalence ratio	*p*-trend
Stroke	(95% CI)	(95% CI)	(95% CI)	
Model 1	Reference	1.126 (0.888~1.428)	1.155 (0.910~1.466)	0.242
Model 2	Reference	1.119 (0.882~1.421)	1.155 (0.908~1.469)	0.247
Model 3	Reference	1.111 (0.875~1.411)	1.146 (0.901~1.458)	0.272
Serum isoleucine levels (mmol/L)				
	Tertile 1 (<0.046)	Tertile 2 (0.046~0.060)	Tertile 3 (>0.060)	
	Prevalence ratio	Prevalence ratio	Prevalence ratio	*p*-trend
Stroke	(95% CI)	(95% CI)	(95% CI)	
Model 1	Reference	1.209 (0.949~1.539)	1.365 (1.077~1.731)	0.010 *
Model 2	Reference	1.204 (0.944~1.534)	1.367 (1.076~1.737)	0.011 *
Model 3	Reference	1.202 (0.943~1.532)	1.352 (1.064~1.718)	0.014 *

Model 1 regulated for age, sex, smoking, and drinking. Model 2 regulated for additional variables such as Townsend deprivation index, mean whole yearly household earning, physical activity, qualifications, and BMI. Model 3 deeply regulated for the hypertension history and the high cholesterol history. * The difference was statistically significant.

## Data Availability

The study utilized UK Biobank data, specifically under application 668944. All the GWAS summary datasets are derived from https://gwas.mrcieu.ac.uk/ (accessed on 1 October 2022). Any additional data analyzed or generated during the research can be found in the published article and supplementary information files.

## Supplementary Materials

The following supporting information can be downloaded at: https://www.mdpi.com/article/10.3390/nu15071580/s1. Table S1: Information of exposure and outcome data sources. Table S2: The total BCAA-related genetic variants used for the MR analyses. Table S3: The Valine-related genetic variants used for the MR analyses. Table S4: The leucine-related genetic variants used for the MR analyses. Table S5: The Isoleucine-related genetic variants used for the MR analyses. Table S6: Baseline characteristics of UK Biobank participants of serum total-BCAA levels. Table S7: Baseline characteristics of UK Biobank participants of serum valine levels. Table S8: Baseline characteristics of UK Biobank participants of serum leucine levels. Table S9: Baseline characteristics of UK Biobank participants of serum isoleucine levels. Table S10: Association between baseline circulating BCAAs in relation to the prevalence of multiple cardiovascular diseases. Table S11: Association between baseline circulating Valine in relation to the prevalence of multiple cardiovascular diseases. Table S12: Association between baseline circulating Leucine in relation to the prevalence of multiple cardiovascular diseases. Table S13: Association between baseline circulating Isoleucine in relation to the prevalence of multiple cardiovascular diseases. Figure S1: Forest plot of cardiovascular diseases causally associated with circulating total BCAAs levels. Figure S2: Forest plot of cardiovascular diseases causally associated with circulating valine levels. Figure S3: Forest plot of cardiovascular diseases causally associated with circulating leucine levels. Figure S4: Forest plot of cardiovascular diseases causally associated with circulating isoleucine levels.
