# Supplementary material for: Association of Circulating Branched-Chain Amino Acids with Cardiovascular Diseases: A Mendelian Randomization Study
Source: Nutrients. 2023 Mar 24;15(7):1580. doi: 10.3390/nu15071580 (PMC10096654; doi:10.3390/nu15071580)
Supplement: Supplementary file 1 [file nutrients-15-01580-s001.zip › nutrients-2292354-supplementary.pdf]

Table S1. Information of exposure and outcome data sources.

Study/Consortium	Exposure/Outcome	Cases	Controls	Sample size	Population	First author (Year)
UK biobank	Total-BCAA	NA	NA	115047	European	Borges CM (2020)
UK biobank	Valine	NA	NA	115048	European	Borges CM (2020)
UK biobank	Leucine	NA	NA	115074	European	Borges CM (2020)
UK biobank	Isoleucine	NA	NA	115075	European	Borges CM (2020)
FinnGen biobank	Peripheral artery disease	7098	206541	213639	European	2021
FinnGen biobank	Transient ischemic attack	8835	202223	211058	European	2021
EBI databases	Small vessel stroke	5386	192662	198048	European	Malik R (2018)
EBI databases	Large artery stroke	4373	406111	410484	European	Malik R (2018)
EBI databases	Cardioembolic stroke	7193	406111	413304	European	Malik R (2018)
EBI databases	Ischemic stroke	34217	406111	440328	European	Malik R (2018)
FinnGen biobank	Subarachnoid hemorrhage	1338	201230	202568	European	2021
FinnGen biobank	Intracerebral hemorrhage	1687	201146	202833	European	2021
FinnGen biobank	Stroke	18661	162201	180862	European	2021
FinnGen biobank	Heart failure	13087	195091	208178	European	2021
EBI databases	Coronary artery disease	42096	361	42457	European	Nikpay M (2015)
MRC-IEU	Angina	18168	187840	206008	European	2021
EBI databases	Myocardial infarction	2680	14125	16805	European	2021

Table S2. The total BCAA-related genetic variants used for the MR analyses.

Exposure	SNP	Chr	Position	Effect allele	Other allele	EAF	Beta	SE	*P* value	Nearby gene	R^2	F-statistic
BCAA	rs1128249	2	165528624	T	G	0.3920	-0.0348	0.0041	2.00E-17	COBLL1	0.000627	72.15
BCAA	rs10018448	4	89225171	G	A	0.5353	0.6965	0.0040	7.29E-128	PPM1K-DT	0.005030	578.67
BCAA	rs2977929	8	76454025	T	C	0.1968	0.0289	0.0050	1.10E-8	HNF4G	0.000284	32.71
BCAA	rs17096421	10	88820592	T	A	0.0564	0.0537	0.0088	9.70E-10	GLUD1	0.000325	37.38
BCAA	rs2638315	12	56865056	C	G	0.1825	0.0439	0.0052	1.80E-17	GLS2, SPRYD4	0.000629	72.36
BCAA	rs36181536	12	122528263	C	T	0.6735	-0.0263	0.0043	7.00E-10	MLXIP	0.000331	38.03
BCAA	rs6499561	16	72148404	G	A	0.3621	0.0239	0.0042	1.30E-08	NA	0.000280	32.26
BCAA	rs117643180	17	7185779	A	C	0.0259	-0.1494	0.0127	3.80E-32	SLC2A4	0.001211	139.29
BCAA	rs12974412	19	14151809	G	A	0.7800	-0.0280	0.0050	1.80E-08	IL27RA	0.000276	31.73
BCAA	rs4801776	19	49304215	T	C	0.2992	-0.0393	0.0045	1.30E-18	BCAT2	0.000674	77.55
BCAA	rs5747934	22	18915282	T	C	0.0423	-0.0050	0.0100	1.20E-09	PRODH	0.000321	36.96

Abbreviations: SNP, single-nucleotide polymorphism; Chr, chromosome; EAF, effect allele frequency; SE, standard error.

Table S3. The Valine-related genetic variants used for the MR analyses.

Exposure	SNP	Chr	Position	Effect allele	Other allele	EAF	Beta	SE	*P* value	Nearby gene	R^2	F-statistic
Valine	rs1128249	2	165528624	T	G	0.3920	-0.0351	0.0041	1.50E-17	NA	0.000631	72.65
Valine	rs10018448	4	89225171	G	A	0.5353	0.1046	0.0040	2.30E-148	PPM1K-DT	0.005849	672.97
Valine	rs12648408	4	89244131	A	G	0.0508	-0.0509	0.0093	4.20E-08	PPM1K-DT	0.000261	30.04
Valine	rs61587941	5	55814890	A	G	0.0512	-0.0502	0.0091	3.40E-08	C5orf67	0.000265	30.45
Valine	rs6941263	6	109162094	A	T	0.1878	-0.0285	0.0052	3.40E-08	NA	0.000265	30.46
Valine	rs17096421	10	88820592	T	A	0.0564	0.0625	0.0088	1.40E-12	GLUD1	0.000436	50.15
Valine	rs36181536	12	122528263	C	T	0.6735	-0.0318	0.0043	1.30E-13	MLXIP	0.000476	54.81
Valine	rs2638315	12	56865056	C	G	0.1825	0.0426	0.0052	2.20E-16	GLS2,SPRYD4	0.000586	67.39
Valine	rs2274815	14	102718052	A	G	0.2050	0.0280	0.0050	1.80E-08	MOK	0.000275	31.68
Valine	rs117643180	17	7185779	A	C	0.0259	-0.1748	0.0127	6.30E-43	SLC2A4	0.001640	188.64
Valine	rs8071084	17	79634162	G	T	0.1617	-0.0323	0.0055	3.10E-09	OXLD1,CCDC137	0.000305	35.11
Valine	rs35230038	19	49300431	A	G	0.0454	-0.0984	0.0098	1.10E-23	BCAT2	0.000875	100.62
Valine	rs117048185	19	49309776	C	G	0.0173	0.1899	0.0154	8.70E-35	BCAT2,LOC105372432	0.001316	151.37
Valine	rs12974412	19	14151809	G	A	0.7800	-0.0272	0.0050	4.80E-08	IL27RA	0.000259	29.81
Valine	rs837616	19	49365588	G	A	0.2915	-0.0250	0.0044	1.60E-08	PLEKHA4	0.000278	31.96
Valine	rs2238732	22	18915347	T	C	0.0423	-0.0621	0.0100	5.80E-10	PRODH	0.000334	38.38

Abbreviations: SNP, single-nucleotide polymorphism; Chr, chromosome; EAF, effect allele frequency; SE, standard error.

Table S4. The leucine-related genetic variants used for the MR analyses.

Exposure	SNP	Chr	Position	Effect allele	Other allele	EAF	Beta	SE	*P* value	Nearby gene	R^2	F-statistic
Leucine	rs11166420	1	100702216	A	T	0.9044	-0.0380	0.0067	1.70E-08	DBT	0.000276	31.77
Leucine	rs13389219	2	165528876	T	C	0.3925	-0.0321	0.0041	2.30E-15	COBLL1	0.000545	62.75
Leucine	rs150277164	4	89258580	A	G	0.0142	0.1159	0.0168	5.20E-12	PPM1K-DT	0.000414	47.62
Leucine	rs10018448	4	89225171	G	A	0.5353	0.0878	0.0040	4.30E-108	PPM1K-DT	0.004239	487.81
Leucine	rs2977929	8	76454025	T	C	0.1968	0.0282	0.0050	1.80E-08	HNF4G	0.000276	31.74
Leucine	rs2638315	12	56865056	C	G	0.1825	0.0409	0.0051	1.20E-15	GLS2,SPRYD4	0.000557	64.06
Leucine	rs9930957	16	72149923	T	C	0.1598	0.0525	0.0054	2.10E-22	NA	0.000824	94.76
Leucine	rs117643180	17	7185779	A	C	0.0259	-0.1184	0.0125	4.00E-21	SLC2A4	0.000773	89.00
Leucine	rs12974412	19	14151809	G	A	0.7800	-0.0291	0.0049	3.30E-09	IL27RA	0.000304	35.01
Leucine	rs4801776	19	49304215	T	C	0.2992	-0.0324	0.0044	2.40E-13	BCAT2	0.000466	53.61
Leucine	rs5747934	22	18915282	T	C	0.0423	-0.0568	0.0099	8.60E-09	PRODH	0.000288	33.14

Abbreviations: SNP, single-nucleotide polymorphism; Chr, chromosome; EAF, effect allel frequency; SE, standard error.

Table S5. The Isoleucine-related genetic variants used for the MR analyses.

Exposure	SNP	Chr	Position	Effect allele	Other allele	EAF	Beta	SE	*P* value	Nearby gene	R^2	F-statistic
Isoleucine	rs10184004	2	165508389	T	C	0.4061	-0.0299	0.0041	4.00E-13	NA	0.000457	52.64
Isoleucine	rs10018448	4	89225171	G	A	0.5353	0.0666	0.0041	4.10E-60	PPM1K-DT	0.002324	267.44
Isoleucine	rs2941456	8	76443463	A	G	0.1985	0.0293	0.0051	9.10E-09	HNF4G	0.000287	33.02
Isoleucine	rs7302925	12	56861458	G	A	0.8011	-0.0413	0.0051	3.60E-16	SPRYD4	0.000577	66.43
Isoleucine	rs12325419	16	70368909	A	G	0.1153	-0.0743	0.0063	1.10E-31	DDX19B,DDX19A-DT	0.001192	137.21
Isoleucine	rs117643180	17	7185779	A	C	0.0259	-0.1014	0.0128	2.90E-15	SLC2A4	0.000542	62.34
Isoleucine	rs545587	19	49319664	C	A	0.5148	0.0366	0.0041	2.30E-19	HSD17B14	0.000704	80.99

Abbreviations: SNP, single-nucleotide polymorphism; Chr, chromosome; EAF, effect allele frequency; SE, standard error.

Figure S1. Forest plot of cardiovascular diseases causally associated with circulating total BCAAs levels.

Outcome	Nsnp	β(95%CI)	P−value	MR−Egger regression	MR−PRESSO global test	Cochran's Q tes
**Peripheral artery disease**	3			0.936	——	0.956
Inverse variance weighted		0.006(−0026~0.039)	0.703			
MR Egger		−0.016(−0.448~0.416)	0.955			
Weighted median		0.005(−0.032~0.041)	0.807			
Simple mode		0.003(−0.040~0.047)	0.893			
Weighted mode		0.004(−0.041~0.048)	0.887			
**Stroke**	4			0.799	0.995	0.992
Inverse variance weighted		0.021(−0.028~0.069)	0.405			
MR Egger		−0.003(−0.168~0.163)	0.976			
Weighted median		0.023(−0.033~0.080)	0.418			
Simple mode		0.024(−0.047~0.095)	0.559			
Weighted mode		0.024(−0.041~0.088)	0.525			
**Angina**	17			0.041	0.086	0.102
Inverse variance weighted		−0.017(−0.041~0.007)	0.257			
MR Egger		−0.066(−0.114~−0.018)	0.227			
Weighted median		−0.013(−0.046~0.019)	0.095			
Simple mode		−0.001(−0.070~0.068)	0.213			
Weighted mode		−0.050(−0.092~−0.008)	0.151			
**Large artery stroke**	4			0.345	0.460	0.411
Inverse variance weighted		0.001(−0.027~0.028)	0.972			
MR Egger		0.052(−0.035~0.138)	0.362			
Weighted median		0.002(−0.306~0.035)	0.887			
Simple mode		0.017(−0.035~0.070)	0.567			
Weighted mode		0.014(−0.034~0.062)	0.600			
**Coronary artery disease**	33			0.139	0.181	0.176
Inverse variance weighted		−0.008(−0.027~0.011)	0.427			
MR Egger		−0.038(−0.080~0.005)	0.095			
Weighted median		−0.007(−0.035~0.021)	0.615			
Simple mode		0.016(−0.044~0.077)	0.598			
Weighted mode		−0.007(−0.051~0.037)	0.750			
**Ischemic stroke**	6			0.442	0.626	0.611
Inverse variance weighted		0.008(−0.044~0.059)	0.764			
MR Egger		0.153(−0.184~0.490)	0.424			
Weighted median		0.029(−0.037~0.096)	0.390			
Simple mode		0.043(−0.060~0.146)	0.448			
Weighted mode		0.045(−0.048~0.137)	0.390			
**Cardioembolic stroke**	4			0.159	0.292	0.170
Inverse variance weighted		−0.006(−0.040~0.028)	0.733			
MR Egger		0.060(−0.004~0.124)	0.210			
Weighted median		0.008(−0.024~0.040)	0.634			
Simple mode		0.003(−0.055~0.060)	0.932			
Weighted mode		0.009(−0.023~0.042)	0.605			
**Myocardial infarction**	24			0.263	0.068	0.057
Inverse variance weighted		0.025(0.000~0.049)	0.051			
MR Egger		−0.014(−0.084~0.056)	0.701			
Weighted median		0.015(−0.016~0.047)	0.344			
Simple mode		0.028(−0.029~0.084)	0.352			
Weighted mode		0.018(−0.018~0.053)	0.348			

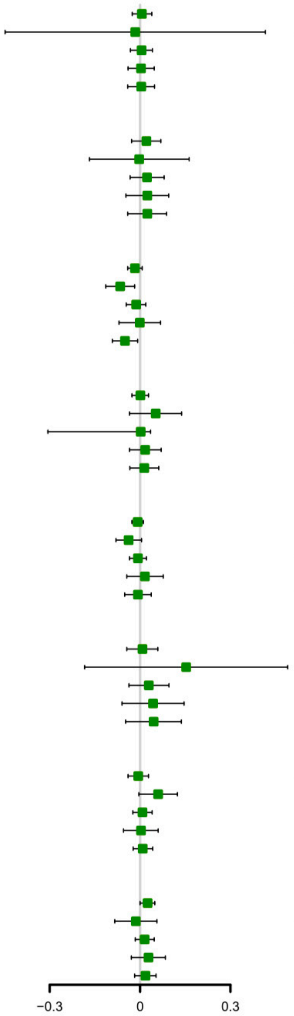

Abbreviations: β, beta value; CI, confidence interval, Nsnp, number of the SNPs.

Figure S2. Forest plot of cardiovascular diseases causally associated with circulating valine levels.

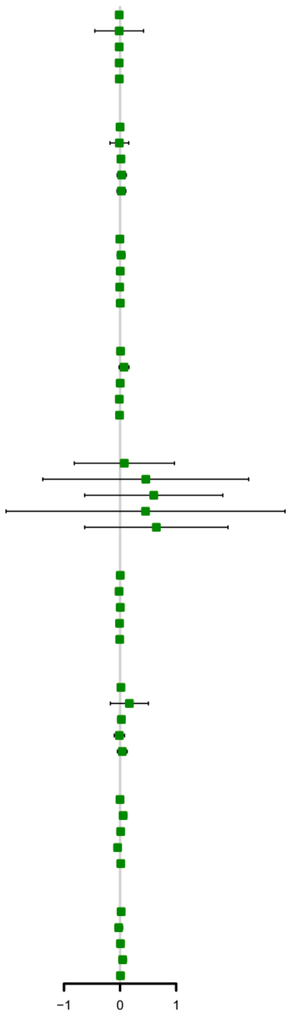

Outcome	Nsnp	β(95%CI)	P−value	MR−Egger regression	MR−PRESSO global test	Cochran's Q test
**Peripheral artery disease**	3			0.998	——	0.886
Inverse variance weighted		−0.015(−0.048~0.018)	0.370			
MR Egger		−0.016(−0.450~0.419)	0.955			
Weighted median		−0.015(−0.054~0.025)	0.464			
Simple mode		−0.016(−0.059~0.027)	0.547			
Weighted mode		−0.014(0.061~0.032)	0.603			
**Stroke**	4			0.887	0.579	0.585
Inverse variance weighted		0.000(−0.049~0.049)	0.994			
MR Egger		−0.013(−0.179~0.154)	0.893			
Weighted median		0.015(−0.043~0.073)	0.615			
Simple mode		0.029(−0.052~0.111)	0.532			
Weighted mode		0.023(−0.055~0.102)	0.599			
**Angina**	16			0.532	0.325	0.294
Inverse variance weighted		−0.004(−0.026~0.018)	0.717			
MR Egger		0.018(−0.054~0.090)	0.625			
Weighted median		0.004(−0.025~0.032)	0.809			
Simple mode		−0.007(−0.055~0.041)	0.770			
Weighted mode		0.005(−0.03~0.039)	0.801			
**Large artery stroke**	4			0.277	0.458	0.379
Inverse variance weighted		0.008(−0.021~0.036)	0.603			
MR Egger		0.070(−0.017~0.157)	0.256			
Weighted median		0.003(−0.030~0.035)	0.869			
Simple mode		−0.013(−0.067~0.041)	0.667			
Weighted mode		−0.009(−0.061~0.042)	0.753			
**Atrial fibrillation**	14			0.647	0.501	0.496
Inverse variance weighted		0.075(−0.817~0.968)	0.869			
MR Egger		0.458(−1.378~2.294)	0.633			
Weighted median		0.601(−0.630~1.833)	0.339			
Simple mode		0.454(−2.033~2.942)	0.726			
Weighted mode		0.647(−0.630~1.925)	0.339			
**Coronary artery disease**	33			0.408	0.185	0.144
Inverse variance weighted		0.004(−0.017~0.025)	0.679			
MR Egger		−0.018(−0.076~0.039)	0.534			
Weighted median		0.005(−0.025~0.035)	0.736			
Simple mode		−0.009(−0.067~0.049)	0.751			
Weighted mode		−0.007(−0.041~0.027)	0.677			
**Ischemic stroke**	6			0.427	0.835	0.832
Inverse variance weighted		0.015(−0.036~0.067)	0.563			
MR Egger		0.166(−0.173~0.505)	0.391			
Weighted median		0.023(−0.040~0.086)	0.468			
Simple mode		−0.013(−0.105~0.079)	0.789			
Weighted mode		0.039(−0.047~0.125)	0.416			
**Cardioembolic stroke**	4			0.203	0.400	0.307
Inverse variance weighted		0.001(−0.030~0.028)	0.923			
MR Egger		0.055(−0.010~0.120)	0.239			
Weighted median		0.010(−0.020~0.041)	0.512			
Simple mode		0.044(−0.100~0.011)	0.218			
Weighted mode		0.012(−0.019~0.043)	0.506			
**Myocardial infarction**	24			0.180	0.092	0.064
Inverse variance weighted		0.019(−0.006~0.044)	0.131			
MR Egger		−0.025(−0.091~0.042)	0.472			
Weighted median		0.007(−0.024~0.039)	0.659			
Simple mode		0.046(−0.018~0.111)	0.170			
Weighted mode		0.007(−0.031~0.045)	0.712			

Abbreviations: β, beta value; CI, confidence interval, Nsnp, number of the SNPs.

Figure S3. Forest plot of cardiovascular diseases causally associated with circulating leucine levels.

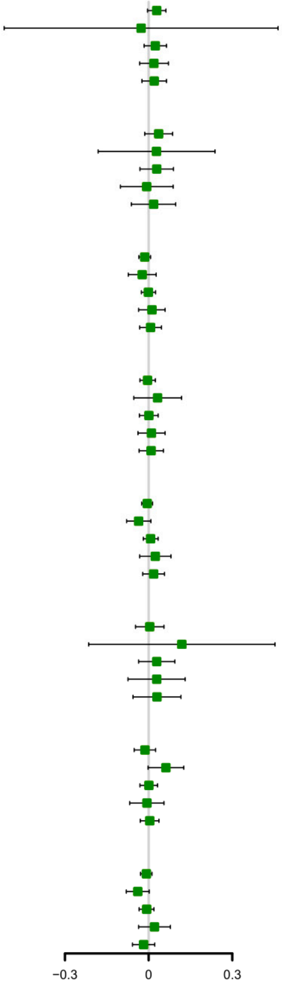

Outcome	Nsnp	ß(95%CI)	P−value	MR−Egger regression	MR−PRESSO global test	Cochran's Q test
**Peripheral artery disease**	3			0.860	——	0.502
Inverse variance weighted		0.029(−0.004~0.062)	0.081			
MR Egger		−0.027(−0.518~0.464)	0.932			
Weighted median		0.024(−0.016~0.064)	0.246			
Simple mode		0.019(−0.032~0.071)	0.533			
Weighted mode		0.020(−0.024~0.064)	0.462			
**Stroke**	4			0.947	0.401	0.353
Inverse variance weighted		0.036(−0.014~0.086)	0.158			
MR Egger		0.028(−0.181~0.238)	0.815			
Weighted median		0.029(−0.031~0.089)	0.339			
Simple mode		−0.007(−0.101~0.088)	0.898			
Weighted mode		0.018(−0.062~0.097)	0.690			
**Angina**	17			0.701	0.142	0.133
Inverse variance weighted		−0.014(−0.035~0.007)	0.202			
MR Egger		−0.023(−0.073~0.027)	0.388			
Weighted median		−0.001(−0.026~0.025)	0.958			
Simple mode		0.012(−0.036~0.059)	0.640			
Weighted mode		0.007(−0.032~0.046)	0.737			
**Large artery stroke**	4			0.456	0.608	0.584
Inverse variance weighted		−0.004(−0.031~0.024)	0.797			
MR Egger		0.032(−0.053~0.118)	0.535			
Weighted median		0.001(−0.033~0.034)	0.971			
Simple mode		0.010(−0.038~0.059)	0.709			
Weighted mode		0.009(−0.034~0.053)	0.700			
**Coronary artery disease**	35			0.135	0.086	0.085
Inverse variance weighted		−0.005(−0.025~0.014)	0.594			
MR Egger		−0.036(−0.079~0.008)	0.116			
Weighted median		0.007(−0.019~0.034)	0.578			
Simple mode		0.024(−0.032~0.080)	0.408			
Weighted mode		0.018(−0.021~0.057)	0.384			
**Ischemic stroke**	6			0.532	0.549	0.531
Inverse variance weighted		0.004(−0.047~0.055)	0.877			
MR Egger		0.119(−0.215~0.453)	0.523			
Weighted median		0.029(−0.036~0.094)	0.384			
Simple mode		0.029(−0.074~0.131)	0.608			
Weighted mode		0.030(−0.056~0.116)	0.524			
**Cardioembolic stroke**	4			0.127	0.235	0.087
Inverse variance weighted		−0.013(−0.052~0.025)	0.495			
MR Egger		0.062(−0.002~0.126)	0.198			
Weighted median		0.001(−0.031~0.032)	0.971			
Simple mode		−0.006(−0.068~0.055)	0.854			
Weighted mode		0.004(−0.03~0.037)	0.849			
**Myocardial infarction**	26			0.109	0.096	0.087
Inverse variance weighted		−0.008(−0.029~0.012)	0.416			
MR Egger		−0.039(−0.080~0.002)	0.074			
Weighted median		−0.007(−0.034~0.019)	0.596			
Simple mode		0.021(−0.036~0.078)	0.476			
Weighted mode		−0.018(−0.058~0.022)	0.393			

Abbreviations: β, beta value; CI, confidence interval, Nsnp, number of the SNPs.

Figure S4. Forest plot of cardiovascular diseases causally associated with circulating isoleucine levels.

Outcome	Nsnp	β(95%CI)	P−value	MR−Egger regression	MR−PRESSO global test	Cochran's Q test
**Peripheral artery disease**	3			0.862	——	0.779
Inverse variance weighted		0.025(−0.008~0.059)	0.139			
MR Egger		−0.024(−0.462~0.414)	0.932			
Weighted median		0.021(−0.016~0.058)	0.267			
Simple mode		0.018(−0.030~0.066)	0.538			
Weighted mode		0.019(−0.026~0.063)	0.496			
**Stroke**	4			0.465	0.475	0.385
Inverse variance weighted		0.050(0.000~0.099)	0.049			
MR Egger		−0.027(−0.202~0.149)	0.794			
Weighted median		0.052(−0.010~0.114)	0.098			
Simple mode		0.067(−0.024~0.158)	0.245			
Weighted mode		0.057(−0.026~0.141)	0.273			
**Angina**	17			0.202	0.202	0.201
Inverse variance weighted		−0.018(−0.038~0.003)	0.094			
MR Egger		−0.046(−0.093~0.004)	0.071			
Weighted median		−0.007(−0.036~0.022)	0.632			
Simple mode		−0.016(−0.039~0.071)	0.578			
Weighted mode		0.014(−0.033~0.061)	0.563			
**Large artery stroke**	4			0.589	0.417	0.361
Inverse variance weighted		−0.012(−0.041~0.017)	0.415			
MR Egger		0.019(−0.082~0.120)	0.747			
Weighted median		−0.001(−0.035~0.034)	0.978			
Simple mode		0.003(−0.045~0.051)	0.912			
Weighted mode		0.002(−0.042~0.046)	0.931			
**Coronary artery disease**	37			0.095	0.010	0.010
Inverse variance weighted		−0.001(−0.022~0.021)	0.947			
MR Egger		−0.039(−0.087~0.010)	0.125			
Weighted median		−0.004(−0.031~0.023)	0.787			
Simple mode		−0.002(−0.053~0.050)	0.946			
Weighted mode		−0.007(−0.042~0.027)	0.683			
**Ischemic stroke**	6			0.563	0.137	0.104
Inverse variance weighted		−0.003(−0.074~0.067)	0.927			
MR Egger		0.153(−0.340~0.646)	0.575			
Weighted median		0.006(−0.071~0.083)	0.874			
Simple mode		−0.040(−0.163~0.083)	0.548			
Weighted mode		0.025(−0.078~0.127)	0.658			
**Cardioembolic stroke**	4			0.153	0.251	0.123
Inverse variance weighted		−0.003(−0.040~0.034)	0.867			
MR Egger		0.065(0.000~0.131)	0.189			
Weighted median		0.012(−0.019~0.043)	0.443			
Simple mode		0.006(−0.046~0.058)	0.829			
Weighted mode		0.012(−0.020~0.044)	0.504			
**Myocardial infarction**	24			0.180	0.064	0.064
Inverse variance weighted		0.019(−0.001~0.044)	0.131			
MR Egger		−0.025(−0.091~0.042)	0.472			
Weighted median		0.007(−0.024~0.038)	0.655			
Simple mode		0.046(−0.012~0.104)	0.130			
Weighted mode		0.007(−0.032~0.046)	0.722			

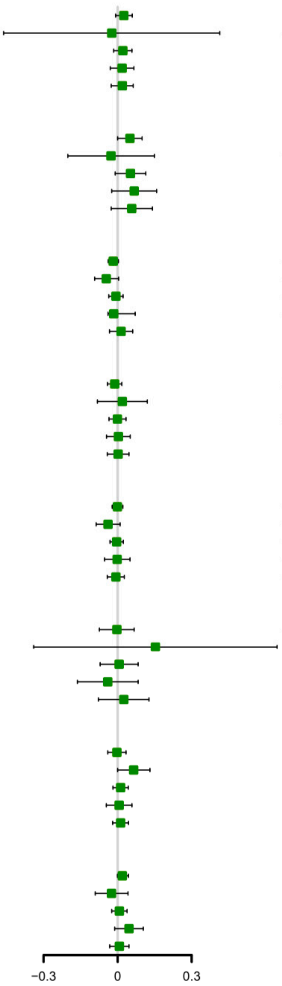

Abbreviations: β, beta value; CI, confidence interval, Nsnp, number of the SNPs.

Table S6. Baseline characteristics of UK Biobank participants of serum total-BCAA levels.

Values are means (SD) or % unless otherwise specified.

	Serum Total-BCAAs levels（mmol/L）			
**Characteristics**	**Tertile 1 (<0.346)**	**Tertile 2 (0.346~0.416)**	**Tertile 3 (>0.416)**	***P*-value**
N	2842	2845	2845	
Age, y	59.45±7.47	59.37±7.12	59.98±7.13	0.031
Male, (%)	26.60	35.20	38.20	<0.001
Drinking status, (%)				0.393
Current	33.10	33.40	33.60	
Previous	34.90	31.00	34.00	
Never	35.00	36.40	28.60	
Missing	40.00	30.00	30.00	
Smoking status, (%)				<0.001
Current	40.50	33.20	26.40	
Previous	30.70	34.00	35.30	
Never	33.70	32.80	33.50	
Missing	33.90	32.10	33.90	
Physical activity, (%)				0.037
Low	33.30	32.90	33.80	
Medium	30.50	33.80	35.70	
High	34.70	34.30	31.00	
Household income (€)				<0.001
<18,000	38.50	31.10	30.40	
18,000~30,999	31.80	33.30	34.90	
31,000~51,999	29.10	35.00	35.90	
52,000~100,000	30.10	35.60	34.30	
>100,000	21.80	37.40	40.80	
Missing	35.40	33.10	31.50	
Qualification				0.049
College or	32.30	33.90	33.80	
University	30.30	33.30	36.40	
A levels/AS levels	33.80	33.10	33.10	
O levels/GCSEs	30.20	31.90	37.90	
CSEs or equivalent	31.30	34.50	34.20	
NVQ/HND/HNC	35.60	33.00	31.40	
None of the above				
BMI, kg/m^2	30.20±5.76	31.77±5.73	32.33±5.42	<0.001
TG, mmol/L	1.30±0.58	1.54±0.61	1.75±0.72	<0.001
TC, mmol/L	4.06±0.97	3.99±0.97	3.92±0.97	<0.001
HDL, mmol/L	1.21±0.32	1.11±0.25	1.09±0.24	<0.001
LDL, mmol/L	1.49±0.45	1.51±0.46	1.48±0.46	0.034
HbA1c, mmol/mol	46.44±12.20	47.88±12.73	50.59±14.32	<0.001
Glucose, mmol/L	6.35±2.41	6.68±2.53	7.59±3.45	<0.001
Creatinine, umol/L	66.36±23.63	69.50±15.25	73.43±15.23	<0.001
Urate, umol/L	320.60±83.62	345.45±80.79	350.62±80.10	<0.001
SBP, mmHg	144.70±18.59	145.00±17.75	144.97±17.69	0.795
DBP, mmHg	82.35±10.66	83.90±10.33	83.64±10.29	<0.001

Abbreviations: BMI, body mass index; TG, triglycerides; TC, total cholesterol; HDL, high-density lipoprotein; LDL, low-density lipoprotein; HbA1c, glycosylated hemoglobin; SBP, systolic pressure; DBP, diastolic pressure.

Table S7. Baseline characteristics of UK Biobank participants of serum valine levels.

	Serum Valine levels（mmol/L）			
**Characteristics**	**Tertile 1 （<0.203）**	**Tertile 2 （0.203~0.238）**	**Tertile 3 （>0.238）**	***P*-value**
N	2844	2844	2843	
Age, y	59.28±7.52	59.58±7.13	58.93±7.06	0.003
Male, (%)	28.20	34.50	37.20	<0.001
Drinking status, (%)				0.130
Current	33.20	33.40	33.40	
Previous	34.90	29.80	35.30	
Never	33.80	37.60	28.60	
Missing	50.00	30.00	20.00	
Smoking status, (%)				<0.001
Current	41.50	32.10	26.40	
Previous	30.70	34.20	35.10	
Never	33.50	32.70	33.80	
Missing	30.40	41.10	28.60	
Physical activity, (%)				0.117
Low	33.60	32.80	33.60	
Medium	31.90	32.50	35.60	
High	33.40	35.10	31.50	
Household income (€)				<0.001
<18,000	38.10	32.30	29.70	
18,000~30,999	31.20	34.50	34.30	
31,000~51,999	30.10	33.20	36.70	
52,000~100,000	30.50	34.20	35.30	
>100,000	22.30	36.30	41.30	
Missing	35.60	32.50	31.90	
Qualification				0.032
College or	32.30	32.70	35.00	
University	29.60	34.30	36.10	
A levels/AS levels	33.90	33.00	33.10	
O levels/GCSEs	32.70	30.70	36.60	
CSEs or equivalent	31.60	35.10	33.30	
NVQ/HND/HNC	35.40	33.60	31.10	
None of the above				
BMI, kg/m^2	29.96±5.64	31.80±5.67	32.56±5.50	<0.001
TG, mmol/L	1.27±0.55	1.54±0.58	1.80±0.74	<0.001
TC, mmol/L	4.02±0.95	3.98±0.99	3.97±0.97	0.116
HDL, mmol/L	1.21±0.32	1.11±0.26	1.10±0.24	<0.001
LDL, mmol/L	1.47±0.44	1.51±0.46	1.50±0.46	0.014
HbA1c, mmol/mol	46.21±11.96	47.95±12.57	50.76±14.61	<0.001
Glucose, mmol/L	6.36±2.41	6.72±2.62	7.54±3.40	<0.001
Creatinine, umol/L	66.71±23.87	69.70±15.08	72.89±15.22	<0.001
Urate, umol/L	320.82±83.52	344.82±80.78	351.06±80.26	<0.001
SBP, mmHg	144.31±18.56	145.14±17.56	145.22±17.90	0.110
DBP, mmHg	82.27±10.71	83.62±10.26	84.00±10.30	<0.001

Values are means (SD) or % unless otherwise specified.

Abbreviations: BMI, body mass index; TG, triglycerides; TC, total cholesterol; HDL, high-density lipoprotein; LDL, low-density lipoprotein; HbA1c, glycosylated hemoglobin; SBP, systolic pressure; DBP, diastolic pressure.

Table S8. Baseline characteristics of UK Biobank participants of serum leucine levels.

	Serum Leucine levels（mmol/L）			
**Characteristics**	**Tertile 1 （<0.096）**	**Tertile 2 （0.096~0.119）**	**Tertile 3 （>0.119）**	***P*-value**
N	2843	2842	2846	
Age, y	59.62±7.35	59.26±7.21	58.92±7.16	0.001
Male, (%)	25.30	35.10	39.60	<0.001
Drinking status, (%)				0.195
Current	32.90	33.40	33.70	
Previous	36.00	31.40	32.60	
Never	37.90	33.80	28.30	
Missing	30.00	30.00	40.00	
Smoking status, (%)				<0.001
Current	38.60	34.10	27.40	
Previous	31.10	33.40	35.50	
Never	34.00	33.00	33.00	
Missing	36.80	35.70	37.50	
Physical activity, (%)				0.206
Low	33.10	33.20	33.70	
Medium	31.60	33.10	35.30	
High	34.70	33.70	31.60	
Household income (€)				<0.001
<18,000	38.00	32.40	29.60	
18,000~30,999	31.60	33.40	35.00	
31,000~51,999	29.90	33.40	36.70	
52,000~100,000	29.90	35.30	34.80	
>100,000	25.10	31.30	43.60	
Missing	35.40	33.50	31.10	
Qualification				0.010
College or	33.60	33.60	32.70	
University	31.10	32.40	36.50	
A levels/AS levels	34.70	31.50	33.80	
O levels/GCSEs	28.00	33.70	38.40	
CSEs or equivalent	29.50	35.90	34.60	
NVQ/HND/HNC	34.70	33.70	31.60	
None of the above				
BMI, kg/m^2	30.54±5.96	31.74±5.73	32.03±5.30	<0.001
TG, mmol/L	1.36±0.62	1.53±0.61	1.71±0.70	<0.001
TC, mmol/L	4.07±0.98	3.99±0.96	3.90±0.97	<0.001
HDL, mmol/L	1.21±0.32	1.12±0.26	1.09±0.24	<0.001
LDL, mmol/L	1.50±0.45	1.51±0.46	1.47±0.45	0.003
HbA1c, mmol/mol	46.92±12.39	47.81±12.23	50.19±13.80	<0.001
Glucose, mmol/L	6.39±2.41	6.65±2.61	7.58±3.40	<0.001
Creatinine, umol/L	66.36±23.63	69.51±15.39	73.42±15.09	<0.001
Urate, umol/L	322.21±83.73	344.47±82.05	350.00±79.22	<0.001
SBP, mmHg	145.19±18.54	144.89±18.17	144.59±18.01	0.450
DBP, mmHg	82.53±10.68	83.84±10.36	83.52±10.27	<0.001

Values are means (SD) or % unless otherwise specified.

Abbreviations: BMI, body mass index; TG, triglycerides; TC, total cholesterol; HDL, high-density lipoprotein; LDL, low-density lipoprotein; HbA1c, glycosylated hemoglobin; SBP, systolic pressure; DBP, diastolic pressure.

Table S9. Baseline characteristics of UK Biobank participants of serum isoleucine levels.

	Serum Isoleucine levels（mmol/L）			
Characteristics	Tertile 1 （<0.046）	Tertile 2 （0.046~0.060）	Tertile 3 （>0.060）	*P*-value
N	2843	2842	2846	
Age, y	59.47±7.37	59.28±7.23	59.05±7.12	0.092
Male, (%)	27.40	34.90	37.80	<0.001
Drinking status, (%)				0.292
Current	33.10	33.50	33.40	
Previous	36.00	29.30	34.70	
Never	34.30	36.00	29.80	
Missing	30.00	40.00	30.00	
Smoking status, (%)				<0.001
Current	38.90	33.60	27.50	
Previous	31.00	33.70	35.30	
Never	33.90	33.00	33.10	
Missing	37.50	26.80	35.70	
Physical activity, (%)				0.447
Low	33.20	33.20	33.60	
Medium	31.50	34.00	34.40	
High	34.60	33.30	32.10	
Household income (€)				<0.001
<18,000	36.90	32.40	30.80	
18,000~30,999	31.80	33.30	34.90	
31,000~51,999	30.80	33.60	35.60	
52,000~100,000	30.80	35.70	33.40	
>100,000	25.70	31.80	42.50	
Missing	35.30	33.10	31.60	
Qualification				0.011
College or	33.20	33.00	33.80	
University	29.60	34.80	35.60	
A levels/AS levels	35.90	30.00	34.10	
O levels/GCSEs	29.70	35.40	34.90	
CSEs or equivalent	31.30	35.00	33.70	
NVQ/HND/HNC	34.00	34.50	31.50	
None of the above				
BMI, kg/m^2	30.45±5.75	31.72±5.70	32.14±5.53	<0.001
TG, mmol/L	1.35±0.61	1.56±0.64	1.69±0.69	<0.001
TC, mmol/L	4.08±0.99	4.01±0.96	3.88±0.96	<0.001
HDL, mmol/L	1.21±0.32	1.12±0.26	1.09±0.24	<0.001
LDL, mmol/L	1.51±0.46	1.51±0.45	1.46±0.45	<0.001
HbA1c, mmol/mol	46.86±12.20	47.91±13.27	50.14±13.94	<0.001
Glucose, mmol/L	6.40±2.40	6.67±2.60	7.56±3.42	<0.001
Creatinine, umol/L	65.89±21.28	69.89±18.32	73.52±15.18	<0.001
Urate, umol/L	324.56±83.28	341.95±82.55	350.18±79.74	<0.001
SBP, mmHg	144.75±18.32	145.23±18.00	144.69±17.71	0.457
DBP, mmHg	82.78±10.52	83.85±10.64	83.25±10.16	0.001

Values are means (SD) or % unless otherwise specified.

Abbreviations: BMI, body mass index; TG, triglycerides; TC, total cholesterol; HDL, high-density lipoprotein; LDL, low-density lipoprotein; HbA1c, glycosylated hemoglobin; SBP, systolic pressure; DBP, diastolic pressure.

Table S10. Association between baseline circulating BCAAs in relation to the prevalence of multiple cardiovascular diseases.

Serum Total BCAAs levels（mmol/L）				
	Tertile1 (<0.346)	Tertile2 (0.346~0.416)	Tertile3 (>0.416)	
**Ischemic stroke**	Prevalence ratio (95% CI)	Prevalence ratio (95% CI)	Prevalence ratio (95% CI)	*p*-trend
Model 1	Reference	1.241 (0.919~1.676)	1.259 (0.931~1.703)	0.143
Model 2	Reference	1.251 (0.924~1.693)	1.267 (0.933~1.721)	0.139
Model 3	Reference	1.248 (0.922~1.689)	1.261 (0.929~1.712)	0.148
**Subarachnoid hemorrhage**				
Model 1	Reference	2.426 (0.440~13.374)	3.364 (0.639~17.693)	0.147
Model 2	Reference	2.338 (0.418~13.063)	3.212 (0.600~17.176)	0.169
Model 3	Reference	2.319 (0.414~12.942)	3.195 (0.596~17.116)	0.171
**Intracerebral hemorrhage**				
Model 1	Reference	1.010 (0.458~2.229)	1.671 (0.817~3.418)	0.132
Model 2	Reference	0.988 (0.445~2.194)	1.647 (0.796~3.407)	0.144
Model 3	Reference	0.998 (0.450~2.215)	1.643 (0.794~3.398)	0.147
**Coronary artery disease**				
Model 1	Reference	1.060 (0.928~1.212)	1.160 (1.015~1.324)	0.028*
Model 2	Reference	1.011 (0.883~1.157)	1.092 (0.954~1.251)	0.193
Model 3	Reference	1.009 (0.881~1.156)	1.083 (0.945~1.241)	0.240
**Myocardial infarction**				
Model 1	Reference	1.207 (0.939~1.551)	1.214 (0.943~1.563)	0.141
Model 2	Reference	1.186 (0.921~1.527)	1.185 (0.918~1.531)	0.207
Model 3	Reference	1.184 (0.920~1.525)	1.182 (0.915~1.527)	0.213
**Peripheral arterial disease**				
Model 1	Reference	1.019 (0.778~1.334)	0.800 (0.600~1.066)	0.130
Model 2	Reference	1.049 (0.798~1.378)	0.832 (0.621~1.113)	0.218
Model 3	Reference	1.047 (0.797~1.377)	0.826 (0.617~1.107)	0.202
**Heart failure**				
Model 1	Reference	1.000 (0.834~1.198)	1.000 (0.832~1.200)	0.996
Model 2	Reference	0.915 (0.761~1.101)	0.899 (0.746~1.085)	0.272
Model 3	Reference	0.912 (0.758~1.097)	0.894 (0.741~1.078)	0.246
**Angina**				
Model 1	Reference	1.080 (0.912~1.280)	1.130 (0.954~1.339)	0.160
Model 2	Reference	1.038 (0.875~1.232)	1.074 (0.905~1.276)	0.414
Model 3	Reference	1.036 (0.873~1.230)	1.067 (0.898~1.267)	0.464

Model 1 adjusted for sex, age, smoking, drinking. Model 2 adjusted for Model 1 and Townsend deprivation index, average total annual household income, physical activity, qualifications, BMI. Model 3 adjusted for Model 2 plus the history of high cholesterol, the history of hypertension.

Table S11. Association between baseline circulating Valine in relation to the prevalence of multiple cardiovascular diseases.

	Serum Valine levels（mmol/L）			
	Tertile1 (<0.203)	Tertile2（0.203~0.238）	Tertile3 (>0.238)	
**Ischemic stroke**	Prevalence ratio (95% CI)	Prevalence ratio (95% CI)	Prevalence ratio (95% CI)	*p*-trend
Model 1	Reference	1.346（1.001~1.809）	1.213（0.894~1.646）	0.233
Model 2	Reference	1.354（1.004~1.827）	1.225（0.898~1.672）	0.224
Model 3	Reference	1.353（1.003~1.825）	1.226（0.898~1.673）	0.223
**Subarachnoid hemorrhage**				
Model 1	Reference	2.348（0.426~12.933）	3.244（0.619~16.986）	0.159
Model 2	Reference	2.259（0.403~12.654）	3.105（0.579~16.661）	0.183
Model 3	Reference	2.219（0.396~12.450）	3.068（0.571~16.486）	0.188
**Intracerebral hemorrhage**				
Model 1	Reference	1.016（0.462~2.237）	1.701（0.835~3.467）	0.119
Model 2	Reference	1.003（0.453~2.223）	1.677（0.810~3.472）	0.133
Model 3	Reference	1.016（0.458~2.252）	1.688（0.815~3.495）	0.130
**Coronary artery disease**				
Model 1	Reference	1.064（0.932~1.216）	1.183（1.036~1.350）	0.012*
Model 2	Reference	1.002（0.876~1.148）	1.099（0.960~1.259）	0.161
Model 3	Reference	1.001（0.874~1.147）	1.100（0.960~1.260）	0.161
**Myocardial infarction**				
Model 1	Reference	1.109（0.863~1.425）	1.217（0.949~1.560）	0.121
Model 2	Reference	1.083（0.841~1.394）	1.182（0.918~1.523）	0.193
Model 3	Reference	1.080（0.838~1.391）	1.181（0.917~1.521）	0.195
**Peripheral arterial disease**				
Model 1	Reference	1.037（0.793~1.356）	0.820（0.615~1.092）·	0.182
Model 2	Reference	1.068（0.814~1.402）	0.858（0.640~1.150）	0.312
Model 3	Reference	1.067（0.812~1.402）	0.860（0.641~1.154）	0.323
**Heart failure**				
Model 1	Reference	0.984（0.820~1.180）	1.084（0.904~1.299）	0.382
Model 2	Reference	0.880（0.731~1.060）	0.950（0.789~1.145）	0.621
Model 3	Reference	0.877（0.728~1.057）	0.950（0.788~1.146）	0.623
**Angina**				
Model 1	Reference	1.107（0.935~1.312）	1.174（0.992~1.390）	0.063
Model 2	Reference	1.054（0.888~1.252）	1.105（0.930~1.313）	0.257
Model 3	Reference	1.053（0.887~1.250）	1.105（0.929~1.313）	0.259

Model 1 adjusted for sex, age, smoking, drinking. Model 2 adjusted for Model 1 and Townsend deprivation index, average total annual household income, physical activity, qualifications, BMI. Model 3 adjusted for Model 2 plus the history of high cholesterol, the history of hypertension.

Table S12. Association between baseline circulating Leucine in relation to the prevalence of multiple cardiovascular diseases.

	Serum Leucine levels（mmol/L）			
	Tertile1 (<0.096)	Tertile2 (0.096~0.119)	Tertile3 (>0.119)	
**Ischemic stroke**	Prevalence ratio (95% CI)	Prevalence ratio (95% CI)	Prevalence ratio (95% CI)	*p*-trend
Model 1	Reference	1.155（0.858~1.554）	1.123（0.831~1.519）	0.463
Model 2	Reference	1.150（0.854~1.551）	1.124（0.830~1.523）	0.464
Model 3	Reference	1.144（0.848~1.541）	1.118（0.825~1.515）	0.485
**Subarachnoid hemorrhage**				
Model 1	Reference	2.458（0.445~13.574）	3.452（0.653~18.253）	0.140
Model 2	Reference	2.352（0.423~13.082）	3.385（0.635~18.061）	0.148
Model 3	Reference	2.330（0.419~12.963）	3.388（0.634~18.114）	0.148
**Intracerebral hemorrhage**				
Model 1	Reference	0.783（0.348~1.761）	1.589（0.791~3.193）	0.143
Model 2	Reference	0.780（0.346~1.761）	1.577（0.780~3.186）	0.150
Model 3	Reference	0.783（0.347~1.766）	1.564（0.774~3.159）	0.157
**Coronary artery disease**				
Model 1	Reference	1.052（0.919~1.203）	1.187（1.039~1.357）	0.011*
Model 2	Reference	1.007（0.879~1.154）	1.137（0.993~1.301）	0.057
Model 3	Reference	0.999（0.872~1.145）	1.126（0.983~1.290）	0.077
**Myocardial infarction**				
Model 1	Reference	1.106（0.862~1.419）	1.104（0.859~1.420）	0.449
Model 2	Reference	1.080（0.841~1.388）	1.081（0.839~1.393）	0.555
Model 3	Reference	1.076（0.838~1.383）	1.079（0.838~1.391）	0.564
**Peripheral arterial disease**				
Model 1	Reference	1.219（0.925~1.607）	0.976（0.730~1.305）	0.837
Model 2	Reference	1.233（0.933~1.629）	1.006（0.750~1.350）	0.995
Model 3	Reference	1.219（0.922~1.612）	1.004（0.747~1.347）	0.984
**Heart failure**				
Model 1	Reference	1.070（0.894~1.281）	0.947（0.786~1.141）	0.562
Model 2	Reference	0.995（0.829~1.195）	0.881（1.055~1.066）	0.188
Model 3	Reference	0.988（0.823~1.187）	0.877（0.726~1.061）	0.173
**Angina**				
Model 1	Reference	1.112（0.937~1.322）	1.240（1.045~1.471）	0.013*
Model 2	Reference	1.073（0.902~1.276）	1.194（1.005~1.419）	0.041*
Model 3	Reference	1.066（0.896~1.268）	1.185（0.998~1.408）	0.051

Model 1 adjusted for sex, age, smoking, drinking. Model 2 adjusted for Model 1 and Townsend deprivation index, average total annual household income, physical activity, qualifications, BMI. Model 3 adjusted for Model 2 plus the history of high cholesterol, the history of hypertension.

Table S13. Association between baseline circulating Isoleucine in relation to the prevalence of multiple cardiovascular diseases.

	Serum Isoleucine levels（mmol/L）			
	Tertile1 (<0.046)	Tertile2 (0.046~0.060)	Tertile3 (>0.060)	
**Ischemic stroke**	Prevalence ratio (95% CI)	Prevalence ratio (95% CI)	Prevalence ratio (95% CI)	*p*-trend
Model 1	Reference	1.017（0.749~1.381）	1.320（0.986~1.766）	0.055
Model 2	Reference	1.013（0.745~1.377）	1.323（0.986~1.775）	0.054
Model 3	Reference	1.012（0.744~1.376）	1.313（0.979~1.763）	0.061
**Subarachnoid hemorrhage**				
Model 1	Reference	7.026（0.841~58.713）	5.120（0.566~46.325）	0.165
Model 2	Reference	6.906（0.822~58.059）	4.979（0.546~45.382）	0.182
Model 3	Reference	6.883（0.818~57.915）	4.988（0.546~45.581）	0.182
**Intracerebral hemorrhage**				
Model 1	Reference	0.866（0.380~1.973）	1.849（0.915~3.736）	0.058
Model 2	Reference	0.863（0.378~1.974）	1.831（0.900~3.725）	0.063
Model 3	Reference	0.865（0.378~1.978）	1.805（0.888~3.670）	0.069
**Coronary artery disease**				
Model 1	Reference	1.085（0.950~1.239）	1.148（1.006~1.311）	0.042*
Model 2	Reference	1.041（0.910~1.191）	1.094（0.957~1.252）	0.188
Model 3	Reference	1.039（0.908~1.190）	1.080（0.943~1.236）	0.265
**Myocardial infarction**				
Model 1	Reference	1.190（0.928~1.526）	1.147（0.892~1.476）	0.299
Model 2	Reference	1.163（0.906~1.492）	1.122（0.870~1.446）	0.393
Model 3	Reference	1.163（0.906~1.493）	1.119（0.868~1.443）	0.403
**Peripheral arterial disease**				
Model 1	Reference	1.106（0.837~1.463）	1.085（0.818~1.440）	0.580
Model 2	Reference	1.113（0.840~1.475）	1.116（0.838~1.485）	0.460
Model 3	Reference	1.113（0.840~1.476）	1.108（0.832~1.476）	0.491
**Heart failure**				
Model 1	Reference	1.126（0.941~1.346）	0.979（0.813~1.178）	0.813
Model 2	Reference	1.050（0.875~1.260）	0.901（0.746~1.089）	0.272
Model 3	Reference	1.048（0.873~1.258）	0.894（0.740~1.080）	0.237
**Angina**				
Model 1	Reference	1.166（0.985~1.380）	1.120（0.945~1.329）	0.204
Model 2	Reference	1.125（0.950~1.333）	1.075（0.905~1.278）	0.432
Model 3	Reference	1.124（0.949~1.333）	1.064（0.896~1.265）	0.507

Model 1 adjusted for sex, age, smoking, drinking. Model 2 adjusted for Model 1 and Townsend deprivation index, average total annual household income, physical activity, qualifications, BMI. Model 3 adjusted for Model 2 plus the history of high cholesterol, the history of hypertension.
